# Supplementary material for: Chiliadenus iphionoides: From chemical profiling to anticancer, antioxidant, α-amylase, and α-glycosidase activities
Source: PLoS One. 2025 Jul 1;20(7):e0327632. doi: 10.1371/journal.pone.0327632 (PMC12212542; doi:10.1371/journal.pone.0327632)
Supplement: Attachment — Submitted filename: CIPHIO~1.DOC [file pone.0327632.s002.DOC]

**Thank you for you valuable Suggestions and comments.**

Response to Editor

1. Please ensure that your manuscript meets PLOS ONE's style requirements, including those for file naming. The PLOS ONE style templates can be found at

**Response: Thank you for your suggestion. Done according to Journal guidelines sections are divided into Heading 1, heading 2 and heading 3 as requested and, Title, References, affiliation all have been edited according to Journal guidelines.**

2. PLOS requires an ORCID iD for the corresponding author in Editorial Manager on papers submitted after December 6th, 2016. Please ensure that you have an ORCID iD and that it is validated in Editorial Manager. To do this, go to ‘Update my Information’ (in the upper left-hand corner of the main menu), and click on the Fetch/Validate link next to the ORCID field. This will take you to the ORCID site and allow you to create a new iD or authenticate a pre-existing iD in Editorial Manager**.**

**Response: Done**

**Response to Reviewers:**

Reviewer #1: There are many studies on the antioxidative and anticancer effects of Chiliadenus iphionoides extracts. In this manuscript, the essential oils were isolated and their chemical content was determined, and the antimicrobial, antioxidative and anticancer effects of this oil were investigated. In this respect, the originality of the study is high.

**Thank you for your comments and suggestions.**

1. The study was well planned and appropriate methods were used. Plant names should be written in italics in the References section.

**Response: Done**

2. In Fig. 3 and 4, A and B should be separate or B should be rearranged.

**Response: Done**, in Fig. 3 and 4, A and B have been separated.

3. Fig. 5 is not of good quality; it should be redrawn and

**Response: Done** Fig. 5 we redraw and rearrange it.

4. In Figs. 6 and 7, the fonts of the x and y axes should be the same.

**Response: Thanks for catching this. Done, both of them are the same font and size.**

**Reviewer #2:** Dear Editor; The attached article was checked. The manuscript contains interesting information about Chiliadenus iphionoides: From Chemical Profiling to Anticancer, Antioxidant, α-amylase, and α-glycosidase activities. I think that this article is well suited to your journal. It is generally good work. The scientific and presentation level of the manuscript is high. The title is understandable and in line with the text. The text is written in a descriptive and understandable language. The material and method are well described and adequately detailed Discussion and conclusion are interrelated.

**Thank you for your comments and suggestions.**

1. **What is the difference between the study and existing studies?**

**Response:**

-This is an excellent question. The plants studied were collected from Jericho, the lowest place on Earth and located very close to the Dead Sea. The soil in Jericho is unique, containing higher salt concentrations compared to other regions. Additionally, the annual precipitation in Jericho is approximately 200 ml. Based on our previous experience, the chemical composition of plants from Jericho differs from that of essential oils collected from other locations.

-Most prior studies have focused on the chemical composition of C. iphionoides essential oil and its antioxidant activity, but only two studies have addressed its anticancer activity. Furthermore, there are currently no studies investigating its α-glucosidase and lipase inhibitory activities. This gap in research presents an opportunity to explore the potential therapeutic benefits of C. iphionoides essential oil. Investigating its effects on α-glucosidase and lipase could reveal valuable insights into its role in managing metabolic disorders and cancer treatment.

- Also, we add a paragraph on the main manuscript line 226-230

2. **add to the introduction, available in different on the wild edible plant studies**

**Response: we added these references as requested. Now they are Ref 4, 5 and 14 in the main and highlighted manuscript.**

**https://doi.org/10.1002/ardp.202300263**

**https://doi.org/10.1002/ardp.202400194**

[**https://doi.org/10.1002/efd2.70021**](https://doi.org/10.1002/efd2.70021)

**Thank you for the suggested references. Done as requested**

3. In ms: Write the name of the authority to the end of the plant’s name.
   - <http://www.theplantlist.org/>
   Please, read the paper and correct them all.

**Response: Done as requested**

4. The paper should be edited according to the writing rules of the journal
   Original manuscript. There are, however, a few minor changes required.

**Response: Done as requested**
